# Antioxidant and Immunostimulatory Activities of a Submerged Culture of *Cordyceps sinensis* Using Spent Coffee

**DOI:** 10.3390/foods10081697

**Published:** 2021-07-22

**Authors:** Sung Hee Han, Yejin Ahn, Hyun Jung Lee, Hyung Joo Suh, Kyungae Jo

**Affiliations:** 1 Institute of Human Behavior & Genetic, College of Medicine, Korea University, Seoul 02841, Korea; sungheeh3@gmail.com; 2 Department of Integrated Biomedical and Life Science, Graduate School, Korea University, Seoul 02841, Korea; cassandra7@hanmail.net (Y.A.); suh1960@korea.ac.kr (H.J.S.); 3 Department of Animal, Veterinary, and Food Sciences, University of Idaho, Moscow, ID 83844, USA; hlee@uidaho.edu; 4 Transdisciplinary Major in Learning Health Systems, Department of Healthcare Sciences, Graduate School, Korea University, Seoul 02841, Korea

**Keywords:** spent coffee grounds, *Cordyceps sinensis*, antiradical, immunostimulatory activity

## Abstract

Spent coffee grounds (SCG) are inexpensive materials that have been used as a source of antioxidants and polysaccharides with immunostimulatory activity. In this study, we performed a microbial fermentation of SCG using *Cordyceps sinensis* and investigated the radical scavenging and immunostimulatory activity of fermented SCG. SCG fermentation using *C. sinensis* was performed at 25 °C for 8 d. The polyphenol content of the fermented SCG increased from 1022.4 to 1562.0 μg/mL. The glucosamine content of the mycelia also continuously increased during fermentation. The main polyphenol compounds of fermented SCG were chlorogenic acid and p-coumaric acid, which were increased by fermentation. Fermented SCG also showed significantly higher content of chlorogenic acid isomers than unfermented SCG. The fermented SCG exhibited significantly higher 2,2-diphenyl-2-picrylhydrazyl hydrate (half maximal inhibitory concentration: IC_50_, 0.37 mg/mL) and 2,2-azinobis (3-ethylbenzothiazoline-6-sulfonic acid) (IC_50_, 0.93 mg/mL) radical scavenging activities than those of the control (0.54 mg/mL and 1.20 mg/mL, respectively; *p* < 0.05). The fermented SCG stimulated macrophages and promoted the production of various immunostimulatory cytokines (IL-12, IL-6, and TNF-α) compared to control; therefore, microbial fermentation of SCG using *C. sinensis* is an effective means of generating antioxidant and immunostimulatory materials.

## 1. Introduction

Coffee is popular due to its unique taste and aroma. In particular, instant coffee is the most popular coffee drink and is consumed by people worldwide. This coffee consumption results in a large amount of residue. Coffee by-products are the wastes extracted from the coffee beans during coffee processing and are classified as coffee pulp, coffee husk, silver skin, and spent coffee grounds (SCG) [1]. Among them, SCG are the main by-product remaining after processing coffee in the coffee beverage market, accounting for 50% of the total coffee bean production, and SCG generation is increasing daily with increases in coffee consumption [2]. Extensive attempts have been made to utilize SCG industrially, such as in active substance extraction [3], mushroom cultivation [4], and bioethanol production [1]. Despite these possibilities, the industrial application of SCG is still insufficient. Growing interest in waste reduction and environment protection have stimulated studies on the possible way of using these waste materials.

Although SCG are widely available and rich in industrially important carbohydrates, proteins, and phenolic compounds [5], they have not been used routinely. SCG are rich in carbohydrates in the form of polymerized cellulose and hemicellulose, comprising nearly half of their dry weight (45.3%, *w*/*w*). Although potential biologically active polyphenols from SCG have rarely been studied, they are believed to be useful. After incomplete thermal extraction of these bioactive components, considerable amounts are speculated to remained in the SCG, which are often dumped as waste.

SCG have been used as an inexpensive source of antioxidants [6] and polysaccharides with immunostimulatory activity [7]. Polyphenols and polysaccharides are reported to have many beneficial health properties owing to their potent antioxidant activity and anti-inflammatory and anticarcinogenic effects [8]. Because of these important biofunctionalities, food and pharmaceutical industries have used the active components in SCG for numerous applications.

The biological conversion method involving fermentation is widely used for industrial applications due to its various advantages, such as the increase of useful ingredients, imparting new physiological activity; increase of absorption; reduction or removal of residual pesticides; increase of useful intestinal microbes; and increase in immune activity when fermentation process is performed [9,10,11]. About 300 species of Cordyceps have been reported worldwide, including the genus Cordyceps belonging to the Clavicipitaceae family of the teleomorph Ascomycetes, as well as *Paecilomyces*, *Torrubiella*, and *Podonectria* [12]. Various physiological activities such as immune-enhancing activity, anticancer activity, antiviral effects, and anti-inflammatory effects have been reported, and the possibility of the use of *Cordyceps sinensis* is increasing [13].

As such, microbes are capable of becoming powerful tools for use in industrial applications of SCG, and *Cordyceps sinensis* could be used for this purpose. Only a few species have been developed for commercial use in the biochemical and medical industries owing to difficulties in elucidating their physiological activity. A submerged culture of SCG was performed using *C. sinensis* to utilize it as a material with enhanced activity, which was selected through a preliminary experiment, after which the radical scavenging and immunostimulatory activity of the SCG fermentation were measured.

## 2. Materials and Methods

### 2.1. Materials

The SCG were collected from a coffee chain franchise (Starbucks) in Korea and dried at 25 °C for 1 d. Caffeine, formic acid, and caffeoylquinic acid (CQA) derivatives (3-, 4-, and 5-CQA) were purchased from Sigma-Aldrich (St. Louis, MO, USA). Folin–Ciocalteu phenol reagent, 2,2-diphenyl-2-picrylhydrazyl hydrate (DPPH), and 2,2-azinobis (3-ethylbenzothiazoline-6-sulfonic acid) diammonium salt (ABTS) were purchased from Sigma Chemical Co. (St. Louis, MO, USA). All the other reagents were obtained commercially from the standard sources.

### 2.2. Defatted SCG and Submerged Culture of C. sinensis

The fat removal of SCG was carried out according to the method described by Choi et al. [14]. The dried SCG were compressed with an expeller (National ENG, Goyang, Korea) at 60 MPa and 100 °C to remove fat, then the residues were used for submerged culture of *C. sinensis*.

For SCG fermentation, *C. sinensis* obtained from Gyeonggi University (Suwon, Korea) was inoculated into 100 mL of potato dextrose broth (Difco Laboratories, Detroit, MI, USA) at an initial pH of 5.5 in a 500-mL flask, then pre-cultured on a rotary shaker (150 rpm) at 25 °C for 7 d. From this initial culture, 4% inoculation was added in a 5 L medium (pH 5.5) containing 300 g of defatted SCG and 10 g of glucose per liter in an 8 L fermenter (Fermentec, Cheongwon, Korea) to obtain the SCG ferment via cultivation for 8 d at 25 °C with 1.0 vvm while stirring at 150 rpm. Fermented SCG was used to measure the antioxidant activity and to prepare exopolysaccharide (EPS).

### 2.3. Analytical Methods

The total polyphenol content was measured using gallic acid as a standard material, according to the method described by Singleton et al. [15]. Reducing sugar and uronic acid were measured using the 3,5-dinitrosalicylic acid [16] and meta-hydroxydiphenyl [17] methods, using glucose and galacturonic acid as standard substances, respectively.

The amount of mycelia during fermentation was measured using a colorimetric method [18] to measure the amount of glucosamine in the mycelia subjected to acid hydrolysis. Hydrolysis of samples (100 mg) was performed in 6 N HCl (5 mL) at 80 °C for 16 h [18]. Total protein was measured using the Bradford method [19] using bovine serum albumin (BSA) as the standard, while 2-keto-3-deoxy-D-manno-octulosonic acid (KDO)-like materials were measured by the modified thiobarbituric acid (TBA) [20]-positive method using 2-keto-3-deoxy-D-manno-octulosonic acid (KDO) as the standard.

For the constituent sugar analysis, EPS was hydrolyzed with 2 M trifluoroacetic acid (TFA) for 90 min at 121 °C [21] and then converted to alditol acetates, which were analyzed by gas chromatography (GC), as reported by Zhao et al. [22].

Polyphenol compounds (gallic acid, 3,4-dihydrobenzoic acid, chlorogenic acid, caffeic acid, p-coumaric acid, trans-ferulic acid, rutin, ellagic acid, quercetin, kaempferol) were analyzed according to the method used by Jo et al. [23] on an high-performance liquid chromatography (HPLC) system (Waters Scientific Ltd., Mississauga, ON, Canada) equipped with a YMC-Triart C18 instrument (4.6 × 250 mm, 5 µm). Mobile phases were analyzed under gradient elution conditions using 0.5% formic acid in water (A) and 0.5% formic acid in acetonitrile (B). The flow rate of the mobile phase was 0.8 mL/min and the sample injection volume was 10 μL, which was detected at 310 nm.

Caffeine and CQA levels were measured using HPLC according to the modified method used by Moon et al. [24]. A Phenomenex Luna C18 column (4.6 × 250 mm, 5 μm) containing 0.2% (*v*/*v*) formic acid in water and 0.2% (*v*/*v*) formic acid in acetonitrile as the mobile phase was used. Caffeine and chlorogenic acid were detected at 276 nm and 325 nm, respectively.

### 2.4. Assay of Radical Scavenging Activity

DPPH, ABTS, and ferric-reducing antioxidant power (FRAP) tests were performed to measure the radical scavenging activity of unfermented and fermented SCG. DPPH radical scavenging activity was expressed as the electron donating activity to DPPH according to the method used by Quang et al. [25]; that is, the sample and the DPPH solution (5 mg/100 mL methanol) were mixed in equal amounts and reacted at 25 °C for 20 min to measure the absorbance at 525 nm. ABTS radical scavenging activity was determined by dissolving potassium persulfate at a concentration of 2.4 mM in 7 mM ABTS solution according to the method used by Wang and Xiong [26], followed by reaction in the dark for 12 to 16 h. The same amount of sample solution was mixed with the ABTS solution and reacted at 25 °C for 10 min to measure absorbance at 415 nm. The radical scavenging ability was expressed as the sample concentration (IC_50_) required to inhibit the generation of radicals by 50%. The FRAP was also analyzed using the method used by Lee et al. [27]. Then, 300 mM acetate buffer (pH 3.6), 10 mM TPTZ (2,4,6-tripyridyl-s-triazine) solution dissolved in 40 mM HCl, and 20 M ferric chloride were mixed at a ratio of 10:1:1 (*v*/*v*/*v*). The mixture was warmed at 37 °C in water bath and used as a FRAP substrate solution. In a 96-well plate, 40 μL of sample solution, 100 μL of FRAP substrate solution, and 40 μL of distilled water were sequentially mixed and reacted at 37 °C. for 4 min, then absorbance was measured at 593 nm.

### 2.5. Preparation of the EPS Fraction

The fermented SCG were filtered using filter paper (Whatman International, Ltd., Maidstone, UK) and centrifuged at 9000× *g* for 20 min. After recovering the supernatant, 4 times the amount of ethanol was added to the volume of the supernatant and stirred, then the mixture was incubated at 4 °C overnight. Thereafter, a precipitate (containing EPS) was obtained by centrifugation at 9000× *g* for 20 min and then lyophilized.

### 2.6. Assay for Cytokine Release from Peritoneal Macrophages

Macrophage activity was measured by slightly modifying the method described by Suzuki et al. [28]. After intraperitoneal injection of 2 mL of 5% thioglycollate medium into BALB/c mice (male, 6–8 weeks old, Coretech) and recovery of the induced macrophages within 72–96 h, the cells were washed 2–3 times with RPMI 1640. The cell number was adjusted to 1 × 10^6^ cells/mL, of which 100 μL was dispensed into a 96-well plate. The cells were then cultured in a 5% CO_2_ incubator (37 °C, 2 h) to form a macrophage monolayer, the supernatant was removed, and the non-adherent macrophages were washed with Rosewell Park Memorial Institute (RPMI) 1640-fetal bovine serum (FBS) medium. Then, the adherent phagocytes were incubated for 24 h with the indicated concentrations of EPS. The respective concentrations of interleukin (IL)-6, IL-12, and tumor necrosis factor (TNF)-α in the culture supernatants were measured with enzyme-linked immunosorbent assay (ELISA) kits (Pharmingen, San Jose, CA, USA).

### 2.7. Statistical Analysis

The experimental results are expressed as the means ± standard deviation (SD), which were obtained using the SPSS statistical program (12.0, SPSS Inc., Chicago, IL, USA), while significance was tested at a significance level of *p* < 0.05 using the Student’s *t*-test.

## 3. Results and Discussion

### 3.1. Changes in Reducing Sugar, Glucosamine, and Polyphenol Contents in a Submerged Culture of C. sinensis with SCG Using an 8 L Fermenter

Fermentation is known to be the most effective method for using food or agricultural by-products. Various degradation products and enzymes can be obtained using carbohydrate-containing by-products as fermentation substrates; therefore, in this study we identified several component changes caused by fermentation. Although no data are displayed, SCG contained 62.1% carbohydrates and 17.6% fat, then after fat removal by compression, SCG contained 68.61% carbohydrates and 8.10% fat (Table S1). Among the carbohydrates, insoluble dietary fiber accounted for 70.1% (Table S2).

**Figure 1:**
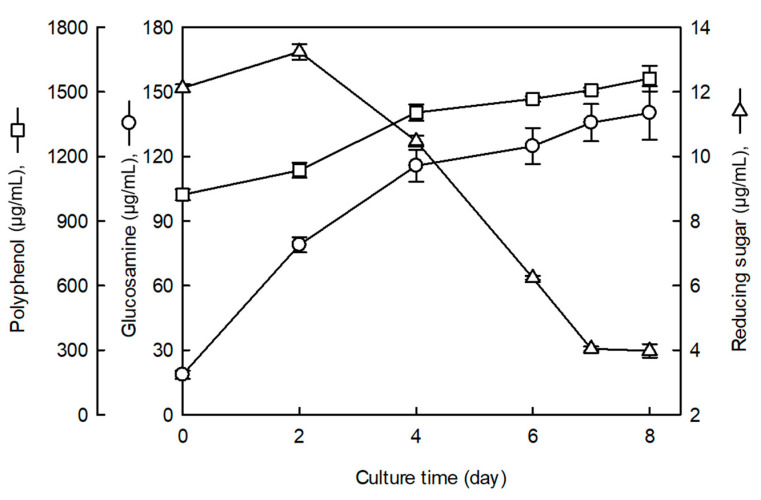
Changes in reducing sugar, polyphenol, and glucosamine contents during the fermentation of spent coffee grounds (SCG) by *C. sinensis*. Values are the means ± SD (*n* = 3).

Figure 1 shows the composition changes during the fermentation of SCG by *C. sinensis* at 25 °C for 8 d. After 2 d of fermentation, the reducing sugar content increased from 12.1 to 13.2 mg/mL, but after 2 d, it rapidly decreased, reaching 3.9 mg/mL on the 8th day. The polyphenol content of the fermented SCG increased from 1022.4 to 1562.0 μg/mL after 8 d of fermentation. Figure 1 also shows a continuous increase in the content of glucosamine in the mycelia during fermentation, from 115.7 μg/mL at 4 d to 140.2 μg/mL at 8 d. As the cell mass of *C. sinensis* increased, the availability of carbohydrates increased, the content of reducing sugar decreased, and the content of polyphenol, an active substance ring contained in coffee meal, increased.

Similarly, fermentation was performed using various mushroom mycelia to increase the SCG solubility. *Pleurotus ostreatus* was reported to decrease in caffeine during cultivation [29], and when *Pleurotus eryngii* was cultured with SCG [30], an increase in mycelium mass was reported.; however, this was the first attempt made to use coffee grounds for the cultivation of *C. sinensis*. The price of the medium plays a significant role in determining the economic feasibility of fermentation products. As cheap and natural products are preferred in most industrial processes, SCG can be selected as a good medium ingredient. SCG is currently used as an animal feed, soil conditioner, and organic fertilizer, and is already known as a source of antioxidants and other nutrients [31].

According to our previous study, *C. sinensis* mycelia extract showed a macrophage-activity-enhancing effect [32], with the production of exopolysaccharides with immune-enhancing activity by *C. sinensis* culture being reported [33]; therefore, the fermented product of *C. sinensis* using SCG is expected to be used as an antioxidant or immune-enhancing material.

### 3.2. Total Sugar, Reducing Sugar, Uronic Acid, and CQA Contents of SCG Fermented Using Submerged Culture of C. sinensis

The total sugar, reducing sugar, uronic acid, and polyphenol contents of SCG after fermentation of *C. sinensis* for 8 d are shown in Table 1. The total contents of sugar (396.27 mg/g) and reducing sugar (217.26 mg/g) for fermented SCG were lower than those of the unfermented control (448.54 and 423.11 mg/g, respectively). Meanwhile, the uronic acid and total polyphenol contents of the fermented SCG were higher than those of the unfermented control. The fermented SCG showed significant differences in total sugar, reducing sugar, uronic acid, and polyphenols from the unfermented SCG (*p* < 0.05, Table 1).

Table 2 shows the changes in polyphenol compounds before and after fermentation of SCG. Table 2 shows the changes in polyphenol compounds before and after fermentation of SCG. Figure S1 shows the HPLC chromatograms before and after fermentation of SCG. Chlorogenic acid was the main polyphenol component of SCG before and after fermentation, showing contents of 2.84 and 9.84 mg/g, respectively (Table 2). Most of the phenolic compounds in coffee exist as caffeic acid, p-coumaric acid, and chlorogenic acid [34]. In the case of SCG, chlorogenic acid and coumaric acid seemed to be the main ingredients, although the amount of caffeic acid was small. The contents of quercetin and kaempferol, which were low or not detected before fermentation, increased after fermentation. It seems that the contents of quercetin and kaempferol increased as glycoside was converted to aglycone via fermentation.

Since chlorogenic acid, the main component of SCG, and caffeine contained in coffee are known to be the main substances for antioxidant activity, these components were analyzed. The caffeine and CQA contents of the fermented SCG are shown in Table 3. CQAs are isomeric forms of chlorogenic acids, which are major phenolic compounds and have many isomeric forms [35]. 3-Caffeoylquinic acid (3-CQA), 4-caffeoylquinic acid (4-CQA), and 5-caffeoylquinic acid (5-CQA) are the major chlorogenic acid isomers in coffee. The results showed that fermented SCG had a significantly higher chlorogenic acid content than the non-fermented control (*p* < 0.001, Table 3). The caffeine content of fermented SCG (29.32 mg/g) was also higher than that of the unfermented control (15.18 mg/g). Chlorogenic acid, which is responsible for the antioxidant activity of coffee, was higher in fermented SCG, indicating beneficial changes in its chemical composition.

Roasting of the coffee resulted in the decomposition of chlorogenic acids into phenolic compounds, which are responsible for its bitterness. The major chlorogenic acids include CQAs, dicaffeoylquinic acids, and feruloylquinic acids. Most studies on the effects of coffee on human health are focused on the negative effects, such as caffeine toxicity [36]; however, recent reports have shown the beneficial effects of brewed coffee, which contains many health-beneficial antioxidants, such as chlorogenic acids [37]. To take advantage of the beneficial effects of drinking coffee, it is important to know which medicinal components, including volatile chemicals and CQAs, are present in coffee [24].

Fermentation is an efficient way to increase the phenolic compound and CQA contents and improve the antioxidant potential of foods. Solid surface fermentation (SSF) by *C. sinensis* alters the chemical composition and biological activity of stale rice [38]. SSF improves the antioxidant activity and nutritional properties of various solid agrowastes [39]. The hydrolytic enzymes produced by fermentation seem to act on coffee grounds to facilitate the release of polyphenols, including CQAs. These enzymes degrade the plant cell wall matrix to promote polyphenol extraction [40]. In addition, *Penicillium purpurogenum*, *Neurospora crassa*, and *Mucor* release large amounts of polyphenols from SCG during solid culture [41].

### 3.3. Antioxidant Activity of Fermented SCG

**Figure 2:**
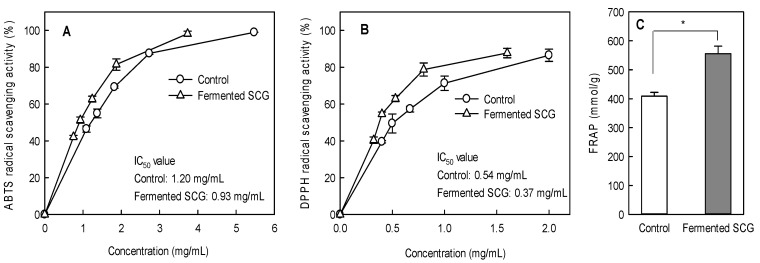
ABTS (**A**) and DPPH (**B**) radical scavenging activity and FRAP (**C**) levels of SCG after 8 days of fermentation. Values are the means ± SD (*n* = 3). The differences between the unfermented (control) and fermented SCG were evaluated using Student’s *t*-test (* *p* < 0.05). The half-maximal inhibitory concentration (IC_50_) is the effective concentration at which 50% of the ABTS or DPPH radical is scavenged.

The antioxidant activities of SCG before and after fermentation are shown in Figure 2. The DPPH (IC_50_, 0.37 mg/mL) and ABTS (IC_50_, 0.93 mg/mL) radical scavenging activities in the fermented SCG were significantly higher than those in the unfermented ones (0.54 mg/mL and 1.20 mg/mL, respectively; *p* < 0.05; Figure 2A,B). The changes in ABTS and DPPH radical scavenging activities showed similar patterns. The fermented SCG also showed significantly higher levels of FRAP (555.57 mmol/g) than the unfermented control (408.88 mmol/g; *p* < 0.05, Figure 2C). We also investigated the chelation power of fermented SCG against Fe^2+^ because it is the most effective pro-oxidant found in the food system [42]. Metal-chelating agents that can prevent the metal-assisted hemolysis of hydroperoxides exert dramatic effects on chain formation.

Fermented SCG had higher uronic acid, polyphenol, and chlorogenic acid contents than the non-fermented SCG (Table 1 and Table 2), which play important roles in the antioxidant activity of fermented SCG. It has been shown that uronic acid, an acidic polysaccharide from plant sources, exhibits many biological activities in addition to antioxidant and immune-stimulating activities [43,44,45]. The total CQA content of coffee is closely related to its ability to scavenge radicals such as peroxyl radicals [46] and chelated iron as natural antioxidants [47]; therefore, fermentation is a very useful process for improving antioxidant activity.

### 3.4. Chemical Properties and Immune-Stimulating Activity of EPS Obtained from SCG in Submerged Culture of C. sinensis

The anticomplementary and radical scavenging activities of a submerged culture of *C. sinensis* were enhanced by the addition of citrus peel [9]. Kuo et al. [48] also reported that EPS from a submerged culture of *C. sinensis* increased its immunological activity. EPS (14.3% yield) from the submerged culture of *C. sinensis* was fractionated by ethanol precipitation and lyophilization to investigate the immunostimulatory activity in the presence and absence of SCG. Although the chemical composition and component sugars of the EPS fraction were not significantly different from each other, a small amount of protein was detected in the no-SCG control, and there was a difference between glucose (*p* < 0.001) and mannose (*p* < 0.01) contents (Table 4); therefore, it seems that the addition of SCG did not significantly affect the EPS composition of *C. sinensis*.

**Figure 3:**
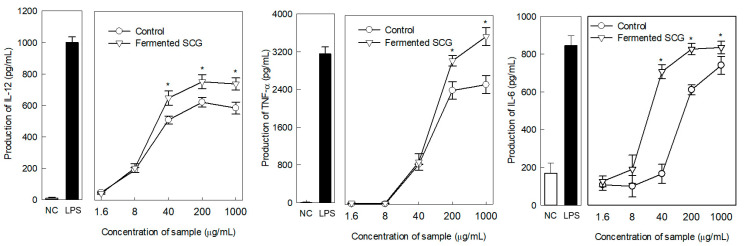
IL-12, TNF-α, and IL-6 production by murine peritoneal macrophages induced by the fermented SCG. Medium was used as the negative control (NC). Lipopolysaccharide (LPS; 5 μg/mL) was used as the positive control. The differences between the unfermented control and fermented SCG were evaluated using Student’s *t*-test (* *p* < 0.05).

The macrophage-stimulating activity of the EPS was significantly higher in the SCG-added culture than in the control, and higher production of all tested cytokines related to macrophage stimulation was observed following the addition of SCG-added culture (Figure 3). The production rates of IL-12 and TNF-α were significantly higher at sample concentrations of 40 and 200 μg/mL or higher, respectively, whereas significant differences in IL-6 were observed at intermediate sample concentrations of 40–200 μg/mL (Figure 3).

IL-6 is a multifunctional cytokine that is important for host defense and is involved in inflammation and infection responses, as well as in the regulation of metabolic, regenerative, and neural processes [49]. IL-6 plays an important role in the regulation of complex cellular processes, such as proliferation, differentiation, inflammation, and immune response [50]. IL-12 is naturally produced by activated antigen-presenting cells (macrophages and dendritic cells) in response to antigenic stimulation [51]. IL-12 is also a powerful inducer of IFN-γ production by T and NK cells while promoting the development of Th1 responses [52]. In addition, TNF-α, a cell signaling protein (cytokine), is involved in systemic inflammation and is produced mainly by activated macrophages. TNF-α plays important roles in the regulation of immune cells, inhibition of tumorigenesis, and viral replication. Dysregulation of TNF-α production has been implicated in various human diseases, including cancer and inflammatory bowel disease [53].

These results suggest that a submerged culture of *C. sinensis* with SCG might stimulate macrophages and promote the production of various immunostimulatory cytokines more than in the control. These results also show that the addition of SCG in a submerged culture is an important factor involved in the formation of EPS with immunostimulatory activity. Further studies should be performed to clarify the structure of EPS involved in immunostimulatory activity when compared to the EPS produced in the control. Based on the results described above, fermentation with *C. sinensis* increased the production of solids, carbohydrates (total sugar, uronic acid, and reducing sugar), and active chlorogenic acid and improved the radical scavenging and immunostimulatory activities of SCG.

## 4. Conclusions

In this study, we performed microbial fermentation of SCG and investigated the radical scavenging and immunostimulatory activities of fermented SCG. The microbial fermentation of SCG changed its chemical properties compared to before fermentation, resulting in increases in antioxidant and immune-stimulating activities. In our results, the increased immune stimulation activity, especially after the fermentation of SCG, was considered to be of great value to allow the use of SCG as functional materials in various fields; therefore, if food by-products are fermented and used as functional food materials, it can be expected that the impacts will be significant, not only in terms of the economic value added, but also for society and public health care.

## Figures and Tables

**Table 1:** Total sugar, reducing sugar, uronic acid, and polyphenol contents in fermented SCG after 8 days of submerged culture.

Sample	Total Sugar (mg/g dw)	Reducing Sugar (mg/g dw)	Uronic Acid (mg/g dw)	Total Polyphenol (mg GAE/g dw)
Control	448.54 ± 17.78	423.11 ± 10.57	7.81 ± 0.52	55.04 ± 1.61
Fermented SCG	396.27 ± 42.14 *	217.26 ± 5.37 *	11.74 ± 0.30 *	81.18 ± 1.38 *

Values are presented as means ± SD (*n* = 3). The differences between the unfermented control and fermented SCG were evaluated using Student’s *t*-test (* *p* < 0.05). GAE: gallic acid equivalent.

**Table 2:** Polyphenol compound contents in fermented SCG after 8 days of submerged culture.

Sample	Content (mg/g dw)			
	3,4-Dihydrobenzoic Acid	Chlorogenic Acid	Caffeic Acid	p-Coumaric Acid
Control	2.90 ± 0.17	1.84 ± 0.03	0.074 ± 0.001	3.65 ± 0.06
Fermented SCG	0.63 ± 0.04 ***	5.04 ± 0.18 ***	0.10 ± 0.004 **	6.52 ± 0.16 ***
Sample	Rutin	Ellagic acid	Quercetin	Kaempferol
Control	0.49 ± 0.003	0.22 ± 0.002	0.56 ± 0.009	n.d.
Fermented SCG	0.66 ± 0.01 ***	0.82 ± 0.007 ***	2.74 ± 0.21 ***	0.49 ± 0.006 ***

Values are means ± SD (*n* = 3). The differences between the unfermented control and fermented SCG were evaluated using Student’s *t*-test (** *p* < 0.01 and *** *p* < 0.001). n.d., not detected.

**Table 3:** Caffeoylquinic acid (CQA) contents in fermented SCG after 8 days of submerged culture.

Sample	Chlorogenic Acid (mg/g dw)			Caffeine(mg/g dw)
	3-CQA	4-CQA	5-CQA	
Control	1.15 ± 0.04	0.63 ± 0.01	0.74 ± 0.02	15.18 ± 0.04
Fermented SCG	4.15 ± 0.06 ***	2.26 ± 0.02 ***	2.71 ± 0.01 ***	29.32 ± 0.01 ***

Values are means ± SD (*n* = 3). The differences between the unfermented control and fermented SCG were evaluated using Student’s *t*-test (*** *p* < 0.001).

**Table 4:** Chemical properties of the crude polysaccharides isolated from the fermented SCG after 8 days of submerged culture.

Coffee Spent (30 g)	Control	Fermented SCG
Chemical composition	Mol%	
Neutral sugar	88.6 ± 6.2	91.9 ± 1.9
KDO-liked material	n.d.	n.d.
Protein	4.1 ± 1.4	n.d.
Sugar component	Mol%	
Rhamnose	0.1 ± 0.1	0.3 ± 0.1
Fucose	n.d.	n.d.
Arabinose	2.2 ± 0.1	3.1 ± 0.5 *
Xylose	n.d.	n.d.
Mannose	47.8 ± 0.5	56.5 ± 1.7 **
Galactose	22.4 ± 0.6	23.6 ± 2.2
Glucose	16.2 ± 0.2	8.4 ± 0.1 ***
GalA + GluA	7.0 ± 0.3	7.6 ± 0.4

KDO = 2-keto-3-deoxy-D-manno-octulosonic acid. Monosaccharides were analyzed using alditol acetates and Mol% was calculated from the detected total carbohydrate content. The differences between the unfermented control and fermented SCG were evaluated using Student’s *t*-test (* *p* < 0.05, ** *p* < 0.01, and *** *p* < 0.001). n.d., not detected.

## Data Availability

The data that support the findings of this study are available from the corresponding author upon reasonable request.

## Supplementary Materials

The following are available online at https://www.mdpi.com/article/10.3390/foods10081697/s1, Figure S1. HPLC chromatogram of polyphenol compounds in control (B) and fermented SCG (C). 1: gallic acid, 2: 3,4-dihydrobenzoic acid, 3: chlorogenic acid, 4: caffeic acid, 5: p-coumaric acid, 6: ferulic acid, 7: rutin, 8: ellagic acid, 9: quercetin, 10: kaempferol Table S1. Proximate composition of spent coffee grounds, Table S2. Dietary fiber composition of spent coffee grounds.
