# Supplementary material for: Antioxidant and Immunostimulatory Activities of a Submerged Culture of Cordyceps sinensis Using Spent Coffee
Source: Foods. 2021 Jul 22;10(8):1697. doi: 10.3390/foods10081697 (PMC8394004; doi:10.3390/foods10081697)
Supplement: Supplementary file 1 [file foods-10-01697-s001.zip › foods-1272343-supplementary.pdf]

Supplementary materials

Table S1. Proximate composition of SCG

Composition (%)	SCG	
	Before fat removal	After fat removal
Moisture	3.67 ± 0.10	4.06 ± 0.06
Ash	1.46 ± 0.22	1.61 ± 0.14
Protein	15.17 ± 0.17	16.77 ± 0.10
Fat	17.62 ± 0.55	8.10 ± 0.25
Carbohydrate	62.08 ± 2.82	68.61 ± 1.03

Values are means ± SD (n=3).

Table S2. Dietary fiber composition of SCG

Dietary fiber	Content (g/100 g)
Total	71.8 ± 1.6
Soluble	1.7 ± 0.0
Insoluble	70.1 ± 1.6

Values are means ± SD (n=3).

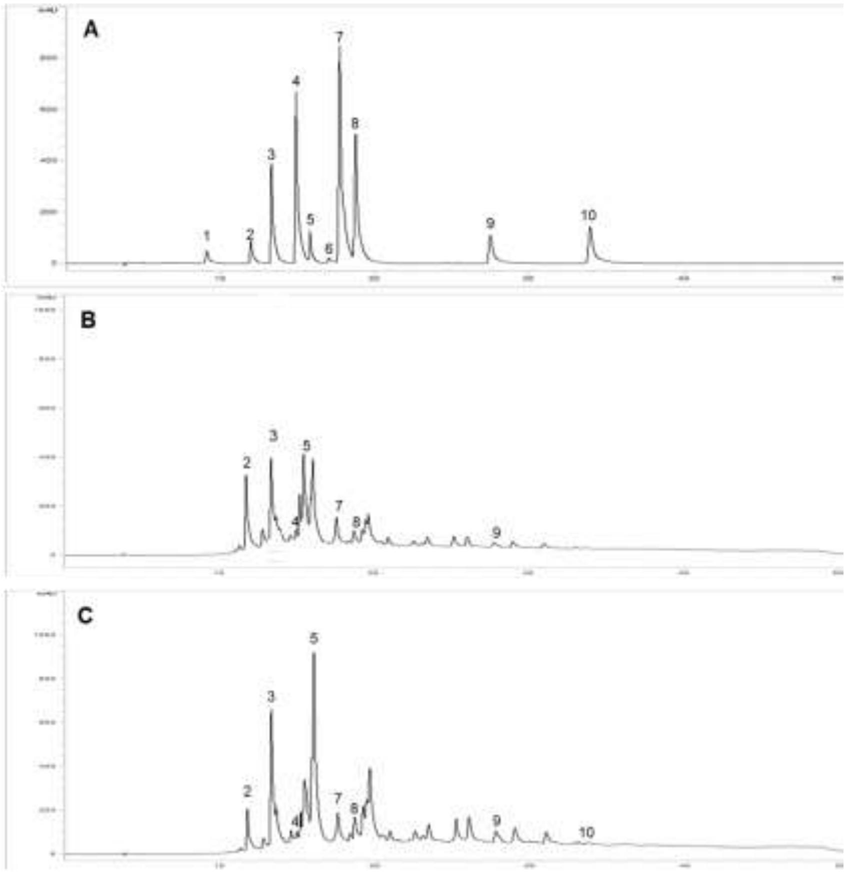

Figure S1. HPLC chromatogram of polyphenol compounds in control (B) and fermented SCG (C). 1: gallic acid, 2: 3,4-dihydrobenzoic acid, 3: chlorogenic acid, 4: caffeic acid, 5: p-coumaric acid, 6: ferulic acid, 7: rutin, 8: ellagic acid, 9: quercetin, 10: kaempferol
